# Bovine tuberculosis and the endangered Iberian lynx.

**DOI:** 10.3201/eid0602.000214

**Published:** 2000

**Authors:** V Briones, L de Juan, C Sánchez, A I Vela, M Galka, Montero, J Goyache, A Aranaz, L Domìnguez

**Affiliations:** Departamento de Sanidad Animal, Facultad de Veterinaria, Universidad Complutense de Madrid, Spain. vbriones@eucmax.sim.ucm.es

## Abstract

We report the first case of bovine tuberculosis in a free-living Iberian lynx (Lynx pardina), an extremely endangered feline, from Doñana National Park in Spain. The isolate (Mycobacterium bovis) correlates by molecular characterization with other isolates from wild ungulates in the park, strongly suggesting an epidemiologic link. Mycobacterium bovis infects many animal species, with wild and free-ranging domestic ungulates being the main reservoirs in nature (1).


### Bovine Tuberculosis and the Endangered Iberian Lynx

**Victor Briones* Lucía de Juan,* Celia Sánchez,† Ana-Isabel Vela,* Margarita Galka,‡ Natalia Montero,* Joaquín Goyache,* Alicia Aranaz,* Ana Mateos,* and Lucas Domínguez***
*Facultad de Veterinaria, Universidad Complutense de Madrid, Spain; †Parque Nacional de Doñana, Huelva, Spain; ‡Parque Natural de Doñana, Huelva, Spain

We report the first case of bovine tuberculosis in a free-living Iberian lynx (*Lynx pardina*), an extremely endangered feline, from Doñana National Park in Spain. The isolate (*Mycobacterium bovis*) correlates by molecular characterization with other isolates from wild ungulates in the park, strongly suggesting an epidemiologic link.

*Mycobacterium bovis* infects many animal species, with wild and free-ranging domestic ungulates being the main reservoirs in nature (1). Carnivores generally acquire the infection by eating infected food (2,3), but reports of tuberculosis (TB) in wild carnivores are rare (2-6). However, TB monitoring in free-living carnivores has provided increased reports of the disease (7). TB does not pose a serious threat to most wild carnivore populations but could have a devastating effect in a small and divided population such as the Iberian lynx. This species is the most endangered feline in the world (8), with a declining number of animals living in reduced and isolated areas in Spain and Portugal.

An adult male Iberian lynx, seen limping in August 1998, died in October 1998. We could perform only direct and radiologic examinations of the empty carcass. The right elbow joint was enlarged, with fistulization. On X-ray, the lesion was diagnosed as septic arthritis on the basis of radiolucent areas and sclerosis, along with bony excrescences. A fragment from the right elbow joint, a small fecal sample, and a nasal swab were collected and routinely processed for detection of mycobacteria by auramine acid-fast staining, culture, and polymerase chain reaction (PCR) (9-11).

Auramine staining of a smear from the elbow lesion was performed by the method of Smithwick (9), and transmission fluorescence microscopy at x400 showed acid-fast bacilli. Samples were simultaneously processed for culture following standard procedures: a suspension was obtained from a nasal swab, and the other samples were homogenized in sterile distilled water in a tissue homogenizer. The homogenates and suspension were then decontaminated with hexadecylpyridinium chloride and centrifuged, and sediments were inoculated onto Coletsos and Löwenstein-Jensen media for mycobacteria; they were incubated at 37°C and inspected weekly for growth (10). Colonies were examined for acid-fast bacilli by the Ziehl-Neelsen technique (9). Identification procedures were performed as described elsewhere, by means of direct PCR either on the processed samples or on isolated colonies (10), and specific mycobacterial molecular characterization was done by spoligotyping (11). The only positive sample was the elbow joint: direct PCR was positive for *M. tuberculosis* complex. An isolate consisting of acid-fast bacilli on selective media was identified as *M. bovis*. Additionally, molecular characterization by spoligotyping showed that the lynx isolate was identical to other isolates from samples of wild ungulates living in the park, including nine wild boars (*Sus scrofa*) and four fallow deer (*Dama dama*) studied during the past 3 years.

Once TB has been confirmed in a free-living lynx, it is important to determine whether a single animal has been affected or the infection has spread within the population. From 1993, postmortem examinations have been performed

Address for correspondence: Lucas Domínguez, Departamento de Sanidad Animal, Facultad de Veterinaria, Universidad Complutense de Madrid, 28040-Madrid, Spain; fax: 34-91-394-3908; e-mail: vbriones@eucmax.sim.ucm.es.

on 21 lynxes in conditions that permitted reliable interpretation of pathologic findings. Any granulomatous lesions found during necropsy, as well as fecal samples taken regularly from temporarily or permanently captive animals, were tested by acid-fast staining, direct PCR, and culture. Radiologic screening has also been performed on captive and live-captured animals.

TB had never been diagnosed in lynxes before this case. However, because of the insidious nature of the infection, this case will not be the last; other lynxes could already be infected. The prevalence of the infection in the species is extremely difficult to determine because the number of lynxes is low and very few animals can be monitored, since captures are limited to a minimum. In addition, rapid, specific, and accurate in vivo diagnosis of TB in lynxes is difficult. A reliable, rapid technique for diagnosis is urgently needed to ensure that any captured animal will be correctly diagnosed and treated.

Another important consideration is the role of wild ungulates and domestic livestock as reservoirs. Populations of red deer (*Cervus elaphus*), fallow deer, and wild boar live in the National Park. Traditional farming in the area means that many domestic animals (mainly free-ranging cattle) coexist with wild species. All these species are susceptible to TB and could act as reservoirs (1). Carnivore populations are generally considered spill-over hosts that become incidentally infected and therefore are unlikely to maintain the disease without an external source of infection (3,6,7). Because eradication campaigns are conducted in domestic cattle, the prevalence of bovine TB is low, but cattle are not TB free. Wild ungulates have been monitored over the last 11 years; a few isolated cases were detected before 1993, although the number of cases has increased dramatically in recent years. This observation may represent a real increase in prevalence or a higher intensity of surveillance. Therefore, surveys of the prevalence of TB among wild ungulates in the Park need to be enhanced to allow a more accurate assessment of the problem.

The isolate of *M. bovis* from the lynx correlates exactly by spoligotyping with isolates obtained recently from wild boars and fallow deer living in the same area, suggesting a common source of infection. As fallow deer are known to be part of the lynx's diet (12), these results are not surprising and strongly suggest interspecies infection. However, since no cultures from cattle are available, the molecular comparison of strains isolated from wild and domestic animals is not possible and a potential epidemiologic link among them cannot be demonstrated. Nevertheless, TB transmission between domestic cattle and wild ungulates sharing the same pastures has been described (13). The different patterns of lesions in red and fallow deer (mainly respiratory) and wild boars (digestive) suggest the probable routes of infection, but the modes of transmission need to be elucidated.

Infectious diseases affecting both domestic and wild animals become a problem for both conservationists and farmers. In this case, the concern is not only transmission between cattle and wild ungulates, but transmission to the Iberian lynx, an endangered species protected by strict conservation policies. Thus, health requirements for domestic animals entering the park should be strengthened to prevent TB, and livestock populations must be managed and controlled, as a total ban seems unfeasible.

The role of lagomorphs in this case is speculative, as TB has not been diagnosed in these species at the park. Although naturally acquired TB seems to be extremely rare in rabbits, it is not uncommon in hares (14). Since these animals are a basic item of the lynx's diet (12), monitoring TB in lagomorphs must be included in a prevention program so that a potential source of infection should not be overlooked.

The detection of bovine TB in an Iberian lynx leads to another question: what should be done with a free-living lynx with a positive TB diagnosis? Intraspecies transmission seems quite unlikely because of the rare contact between lynxes except during mating season. However, basic rules of animal health dictate that infected animals should be isolated to interrupt the transmission cycle of many infectious diseases, including TB.

Anti-TB therapy in animals is controversial, and euthanasia is the most commonly accepted option. However, the critical situation of the Iberian lynx, with a declining population of under 1,000, makes every animal essential. Multidrug, long-term, daily therapy has been used in domestic cats (2,3), but extrapolating this treatment to lynxes maintained in captivity would be difficult. Although therapy must be the choice, adequate facilities and containment and preventive measures must be in place to ensure that disease will not spread during treatment. In addition, the likelihood of success of treatment, which depends on the severity of the disease and the general condition of the animal, must be evaluated in light of the well-known collateral effects of antimycobacterial drugs (2,3). Finally, even if treatment is successful, the animals will probably not be able to live in the wild again because of possible physical or behavioral changes.

In conclusion, survival of many species already depends on human management of populations (15); in this case, every measure must be adopted to ensure that bovine TB will not threaten the endangered Iberian lynx.

Dr. Briones is associate professor, Departamento de Sanidad Animal, Facultad de Veterinaria, Universidad Complutense de Madrid. His responsibilities include clinical microbiology and infectious diseases (research and teaching). His research interests focus on bacterial diseases of wildlife and exotic pets in Spain.

### References

1. Hunter DL. Tuberculosis in free-ranging, semi free-ranging and captive cervids. Rev Sci Tech 1996;15:171-81.
2. Gunn-Moore DA, Jenkins PA, Lucke VM. Feline tuberculosis: a literature review and discussion of 19 cases caused by an unusual mycobacterial variant. Vet Rec 1996;138:53-8.
3. Greene CE, Gunn-Moore DA. Tuberculous mycobacterial infections. In: Greene CE, editor. Infectious diseases of dog and cat. 2nd ed. Philadelphia (PA): WB Saunders; 1998. p. 313-21.
4. Helman RG, Russell WC, Jenny A, Miller J, Payeur J. Diagnosis of tuberculosis in two snow leopards using polymerase chain reaction. J Vet Diagn Invest 1998;10:89-92.
5. Thorel MF, Karoui C, Varnerot A, Fleury C, Vincent V. Isolation of *Mycobacterium bovis* from baboons, leopards and a sea-lion. Vet Res 1998;29:207-12.
6. Artois M, Claro F, Remond M, Blancou J. Infectious pathology of Canidae and Felidae in zoological parks. Rev Sci Tech 1996;15:115-40.
7. Keet DF, Kriek NPJ, Penrith M-L, Michel A, Huchzermeyer H. Tuberculosis in buffaloes (*Syncerus caffer*) in the Kruger National Park: spread of the disease to other species. Onderstepoort J Vet Res 1996;63:239-44.
8. Nowell K, Jackson P, editors. Wild cats: status survey and conservation action plan. IUCN Publications. Cambridge: The Burlington Press; 1996.
9. Smithwick RW, editor. Laboratory Manual for acid-fast microscopy. 2nd ed. Georgia: U.S. Department of Health, Education and Welfare; 1976.
10. Aranaz A, Liebana E, Pickering X, Novoa C, Mateos A, Dominguez L. Use of polymerase chain reaction in the diagnosis of tuberculosis in cats and dogs. Vet Rec 1996;138:276-80.
11. Kamerbeek J, Schouls L, Kolk A, van Agterveld M, van Soolingen D, Kuijper S, et al. Simultaneous detection and strain differentiation of *Mycobacterium tuberculosis* for diagnosis and epidemiology. J Clin Microbiol 1997;35:907-14.
12. Delibes M. Feeding ecology of the Spanish lynx in the Coto Doñana. Acta Theriologica 1980;25:309-24.
13. Tweddle NE, Livingstone P. Bovine tuberculosis control and eradication programs in Australia and New Zealand. Vet Microbiol 1994;40:23-39.
14. Gill JW, Jackson R. Tuberculosis in a rabbit: a case revised. New Zealand Veterinary Journal 1993;41:147.
15. Kirkwood JK. Interventions for wildlife conservation, health and welfare. Vet Rec 1993;132:235-8.
